# Targeted Reinnervation During Gender-Affirming Mastectomy and Restoration of Sensation

**DOI:** 10.1001/jamanetworkopen.2024.46782

**Published:** 2024-11-22

**Authors:** Katya Remy, Chase Alston, Elyse Gonzales, Merel H. J. Hazewinkel, Katherine H. Carruthers, Leslie E. Cohen, Eleanor Tomczyk, Jonathan M. Winograd, William G. Austen, Ian L. Valerio, Lisa Gfrerer

**Affiliations:** 1 Division of Plastic and Reconstructive Surgery, Department of Surgery, Massachusetts General Hospital, Harvard Medical School, Boston; 2 Division of Plastic and Reconstructive Surgery, Department of Surgery, Weill Cornell Medicine, Weill Cornell Medical College, New York, New York

## Abstract

**Importance:**

During gender-affirming mastectomy, nerves are transected, resulting in sensory loss. Nerve preservation using targeted nipple-areola complex (NAC) reinnervation (TNR) may restore sensation.

**Objective:**

To determine the quantitative and patient-reported sensory outcomes of TNR.

**Design, Setting, and Participants:**

Prospective matched cohort study of patients undergoing gender-affirming mastectomy from August 2021 to December 2022 at Weill Cornell Medicine and Massachusetts General Hospital. Data were analyzed from January to March 2023.

**Exposure:**

Patients who underwent TNR and matched patients who did not.

**Main Outcomes and Measures:**

Mechanical detection measured with monofilaments and patient-reported outcome questionnaires were completed preoperatively and at 1, 3, 6, 9, and 12 months postoperatively. Additional quantitative sensory testing was performed preoperatively and at 12 months postoperatively. The primary outcome was mechanical detection while secondary outcomes were the additional quantitative sensory testing variables and patient-reported outcomes. Exclusion criteria included peripheral nerve disorders, unmatched patients, and incomplete follow-up.

**Results:**

A total of 25 patients who underwent TNR and 25 matched patients who did not were included. The mean (SD) age was 24.9 (5.5) years, BMI was 26.6 (5.2), and mastectomy weight was 608.9 (326.5) g; 6 patients (12.0%) were Asian, 5 patients (10.0%) were Black or African American, and 33 patients (66.0%) were White. Repeated measures analysis of variance (ANOVA) showed that the outcomes of TNR on improving mechanical detection over time was significant at the NAC (*F* = 35.2; *P* < .001) and chest (*F* = 4.2; *P* = .045). At 12 months, mean quantitative sensory values in patients who underwent TNR reached baseline and were improved compared with patients who did not undergo TNR for monofilaments (mean [SD] NAC, 3.7 [0.5] vs 4.9 [0.9]; [data]; *P* < .001; chest, 3.3 [0.4] vs 3.6 [0.6]; [data]; *P* = .002), vibration (mean [SD] NAC, 7.7 [ 0.4] vs 7.3 [0.4]; *t*_96_ = 6.3; *P* < .001; chest, 7.8 [0.3] vs 7.5 [0.3]; *t*_96_ = 5.1; *P* < .001), 2-point discrimination (NAC, 40% vs 0%; *r* = 20; *P* = .02); chest, 4.1 [1.2] cm vs 5.7 [1.8] cm; *P* < .001), pinprick (mean [SD] NAC, 24.9 [21.2] mN vs 82.6 [96.7] mN; *t*_98_ = 4.1; *P* < .001; chest, 22.5 [25.6] mN vs 54.1 [45.4] mN; *t*_98_ = 4.6; *P* < .001), cold (mean [SD] NAC, 23.1 [4.7] °C vs 12.0 [7.6] °C; *t*_98_ = 8.8; *P* < .001; chest, 23.6 [3.1] °C vs 19.7 [5.6] °C; *t*_98_ = 4.4; *P* < .001), warm (mean [SD] NAC, 39.9 [5.0] °C vs 45.8 [4.2] °C; *t*_98_ = 6.3; *P* < .001; chest, 39.4 [3.1] °C vs 42.9 [4.0] °C; *t*_98_ = 4.9; *P* < .001), and pressure pain detection (mean [SD] NAC, 89.9 [45.6] kPa vs 130.5 [68.9] kPa; *t*_86_ = 3.9; *P* < .001; chest, 128.5 [38.0] kPa vs 175.5 [49.3] kPa; *t*_96_ = 4.0; *P* = .001). ANOVA demonstrated that TNR significantly improved patient-reported nipple sensation (*F* = 60.5; *P* < .001), chest light touch (*F* = 8.1; *P* = .01) and erogenous sensation (*F* = 8.3; *P* = .01). Significantly more patients who underwent TNR than those who did not reported nipple hypersensitivity until 3 months postoperatively (8% vs 4% at 12 months).

**Conclusion and Relevance:**

In this prospective matched cohort study, TNR was associated with improved quantitative and patient-reported sensation. Patients should be counseled about the risk of transient NAC hypersensitivity.

## Key Points

**Question:**

Can nerve preservation and reconstruction using targeted nipple-areola complex reinnervation (TNR) improve sensory outcomes following gender-affirming mastectomy?

**Findings:**

In this cohort study that included 50 patients undergoing gender-affirming mastectomy with free nipple grafts, TNR was associated with improved quantitative and patient-reported sensory function, including mechanical, 2-point discrimination, vibration, pinprick, temperature detection, heat pain detection, pressure pain detection, and erogenous sensation as compared with control patients who did not undergo TNR.

**Meaning:**

These findings suggest that in patients undergoing gender-affirming mastectomy, TNR allowed for improved restoration of sensation.

## Teaser

This cohort study evaluates the quantitative and patient-reported sensory outcomes of targeted reinnervation during gender-affirming mastectomy.

## Introduction

Gender-affirming mastectomy is the most commonly performed gender-affirming care procedure in the US, representing 85% of procedures performed in patients transitioning from female to either male or nonbinary.^1,2^ Of the various techniques that exist, double incision mastectomy with free nipple grafting is the most frequent procedure as it allows for the removal of large volumes of breast tissue to achieve a flat chest.^3,4,5^

Loss of sensation has been reported to be a common risk associated with gender-affirming mastectomy.^6^ This risk is highest with the double incision technique since the intercostal nerves that travel through the breast tissue to provide nipple-areola complex (NAC) and chest sensation are transected. Studies on gender mastectomy have found that most patients report loss of nipple sensation following surgery.^6,7^ In a study^8^ including 95 patients undergoing the double incision technique, loss of nipple sensation was reported in 100% of patients.

The broader implications of sensory loss have been mainly studied in cancer mastectomy and breast reduction surgery patients, showing significant consequences on functional and psychological well-being, including a heightened risk of injury and reduced quality of life.^9,10,11,12,13,14,15,16^ These studies have not been performed in the transgender and nonbinary population undergoing gender-affirming mastectomy. However, the majority of patients report that sensation is important to them preoperatively, and loss of sensation significantly reduces satisfaction postoperatively.^14,17^

Restoration of sensation through chest reinnervation is a growing field of interest due to the increased awareness of unreliable sensory outcomes following gender-affirming mastectomy. Our group described targeted NAC reinnervation (TNR), a technique to preserve and reconstruct all encountered intercostal nerves to maximize sensory recovery.^18^ In a recent prospective cohort study of 25 patients undergoing gender-affirming mastectomy with TNR, we found that mechanical detection through monofilament testing was restored to baseline sensation within 3 months postoperatively at the NAC and chest, with a high satisfaction rate at 12 months postoperatively.^19^

Although the current evidence on reinnervation techniques to improve sensation is promising, only monofilament testing has been evaluated in previous studies.^19,20^ Other quantitative somatosensory functions such as vibration, 2-point discrimination, pressure, temperature, and pain have not been analyzed. In addition, there is little evidence on patient-reported sensory outcomes, including erogenous sensation. Therefore, this study aims to comprehensively analyze the various sensory outcomes following gender-affirming mastectomy with TNR using a validated and standardized method of quantitative sensory testing, as well as patient-reported outcomes in a prospective matched cohort study design.

## Methods

### Study Design and Patient Cohort

This multicenter prospective matched cohort study received institutional review board approval at Weill Cornell Medical Center and Massachusetts General Hospital. Patients who consulted for double-incision gender-affirming mastectomy with free nipple grafting were recruited at both institutions between August 2021 and December 2022. Patients were presented with the option to undergo TNR at the time of their consultation. Inclusion criteria included being aged 18 years or older, fulfillment of the World Professional Association for Transgender Health requirements for masculinizing chest surgery, and willingness and ability to provide oral informed consent. Exclusion criteria included a history of neurological or peripheral nerve disorders and previous breast surgery. Self-reported race and ethnicity (American Indian or Alaska Native, Asian, Black or African American, White, or Other) were collected through medical records to assess the generalizability of the findings based on the study population. The study followed the Strengthening the Reporting of Observational Studies in Epidemiology (STROBE) reporting guideline for observational studies.

### Surgical Technique

In patients undergoing gender-affirming mastectomy with TNR, the procedure was performed as previously described.^18^ In short, intercostal nerve branches encountered during the mastectomy procedure were identified and preserved at a length that could reach the NAC directly. In cases where branches could not be preserved at sufficient length, an acellular Avance nerve allograft (Axogen) was used. At the level of the NAC, intercostal nerve branches and allografts were split distally into fascicles and fanned out underneath the free nipple grafts (eFigure 1 in Supplement 1). The free nipple graft dimensions were 2.2 cm by 2.5 cm.

### Quantitative Sensory Testing

Quantitative sensory testing was performed following the German Research Network on Neuropathic Pain protocol.^21^ All sensory testing was performed on the NAC and surrounding chest skin on predefined quadrants as depicted in eFigure 2 in Supplement 1.

Semmes-Weinstein monofilament testing was performed to assess mechanical detection threshold preoperatively and postoperatively at 1, 3, 6, 9, and 12 months. Monofilament index values included 2.83, 3.61, 4.34, 4.56, and 6.65.

Additional sensory testing was performed preoperatively and postoperatively at 12 months. Two-point discrimination was recorded as the minimum distance where 2 points on the device could be distinguished by the patient. Vibration detection threshold was measured using a 128Hz tuning fork with a scale ranging from 0 (no vibration detection) to 8 (highest vibration sensitivity). Cold, warm and heat pain detection thresholds were assessed using a thermal sensory testing device with a 15 mm by 15 mm contact area probe (TSA2, MEDOC). From a baseline temperature set to 32 °C, the probe progressively cooled to a minimum temperature of 0 °C or warmed to a maximum temperature of 50 °C. Patients were asked to report when they started to feel cold, warmth, and then warmth associated with discomfort. Pinprick index values included 8, 16, 32, 64, 128, 256, and 512 mN, and patients were asked to communicate when the instrument felt sharp upon application (PinPrick, MRC Systems). Pressure pain detection threshold was assessed using an algometer with a 10 mm by 10 mm contact area and with a 10 kg/1000 kPa/100 N applicable force (Algomed, MEDOC). Patients were asked to communicate when the applied pressure became uncomfortable.

### Patient-Reported Outcomes

Questions were generated by the investigator team based on clinical experience given that no validated patient-reported outcome measures for chest sensory outcomes exist for transgender patients. Preoperatively, patients were asked to rate their level of concern for sensation, including light touch and protective and erogenous sensation (1 = not at all concerned, 2 = slightly concerned, 3 = somewhat concerned, 4 = very concerned, or 5 = extremely concerned). Postoperatively at 1, 3, 6, 9, and 12 months, patients were asked to rate nipple sensation and chest light touch, chest pressure, the ability to feel cold, the ability to feel warm, the ability to feel a hug, the ability to feel water from the shower, erogenous sensation, and nipple erection (1 = none, 2 = a little, 3 = some, 4 = a lot, or 5 = complete). Satisfaction with nipple and chest sensation was determined at 12 months postoperatively (1 = extremely dissatisfied, 2 = very dissatisfied, 3 = somewhat dissatisfied, 4 = somewhat satisfied, 5 = very satisfied, or 6 = extremely satisfied). The full verbatim text of questions can be found in the eTable in Supplement 1. Data were recorded on REDCap version 8.1.20 (Vanderbilt University). Adverse events were collected retrospectively through medical record review and included any unanticipated event including acute and delayed hematoma, partial or complete free nipple graft necrosis, mastectomy flap necrosis, seroma, acute and chronic infection, wound dehiscence, hypertrophic scarring, and nipple hypopigmentation documented in clinical notes.

### Statistical Analysis

Patients who underwent TNR and those who did not were matched 1:1 by age (±5 years), body mass index (BMI) (±5; calculated as weight in kilograms divided by height in meters squared) and mastectomy weight (±250 g) at the time of surgery, given that these demographic variables have been shown to influence chest sensation.^22,23^ The degree of ptosis was not matched on given that this variable was not consistently recorded preoperatively. The primary outcome analysis consisted of measuring the outcomes of TNR on mechanical detection threshold over postoperative time using repeated measures analysis of variance (ANOVA). Secondary outcome analyses consisted of evaluating additional quantitative sensory testing variables (vibration, 2-point discrimination, pinprick, cold detection, warm detection, heat pain, and pressure pain), patient-reported outcomes *t* test, and repeated measures ANOVA. Each side of the chest was independently evaluated. Continuous variables were described using means and SDs or medians and IQRs depending on normality. Categorical variables were described using frequencies and percentages. A power analysis based on the expected effect size, an α level of .05, and a desired power of 0.80 determined 50 patients to be the required sample size to detect significant differences or associations. A 2-sided *P* value less than .05 was considered statistically significant. Statistical analyses were performed with John’s Macintosh Project version 16 (SAS Institute). Data were analyzed from January to March 2023.

## Results

### Patient Demographics and Characteristics

A total of 120 patients were enrolled. Of these, 25 patients who underwent gender-affirming mastectomy with TNR were compared with 25 age-, BMI-, and mastectomy weight-matched control patients who underwent gender-affirming mastectomy without TNR. A total of 70 patients were excluded, including 33 who did not undergo surgery, 28 due to incomplete 12 month follow-up, 6 who were not matched, and 3 with a history of peripheral nerve disorders. The mean (SD) age of the study population was 24.9 (5.5) years, the mean (SD) BMI was 26.6 (5.2), and the mean (SD) mastectomy weight was 608.9 (326.5) g; 6 patients (12.0%) were Asian, 5 patients (10.0%) were Black or African American, and 33 patients (66.0%) were White. Patient demographics were not significantly different between patients who underwent TNR and those who did not (see Table).

**Table.** Patient Demographics

Variable	Patients, No. (%)			*P* value
	Total (N = 50)	Underwent TNR (n = 25)	Did not undergo TNR (n = 25)	
Age, mean (SD), y	24.9 (5.5)	24.3 (5.3)	25.7 (5.7)	.39
BMI, median (SD)^a^	26.6 (5.2)	25.5 (4.1)	27.7 (6.0)	.13
Mastectomy weight, median (SD), g	608.9 (326.5)	587.8 (362.8)	630.1 (287.9)	.52
Race				
American Indian/Alaska Native	1 (2.0)	1 (4.0)	0	
Asian	6 (12.0)	3 (12.0)	3 (12.0)	.55
Black or African American	5 (10.0)	3 (12.0)	2 (8.0)	
White	33 (66.0)	17 (68.0)	16 (64.0)	
Other^b^	5 (10.0)	1 (4.0)	4 (16.0)	
Ethnicity				
Hispanic or Latino	9 (18.0)	4 (16.0)	5 (20.0)	<.99
Not Hispanic or Latino	41 (82.0)	21 (84.0)	20 (80.0)	

^a^ Calculated as weight in kilograms divided by height in meters squared.

^b^ Indicates participants who chose “other” as their race.

### Quantitative Sensory Testing

#### Mechanical Detection Threshold

**Figure 1.**
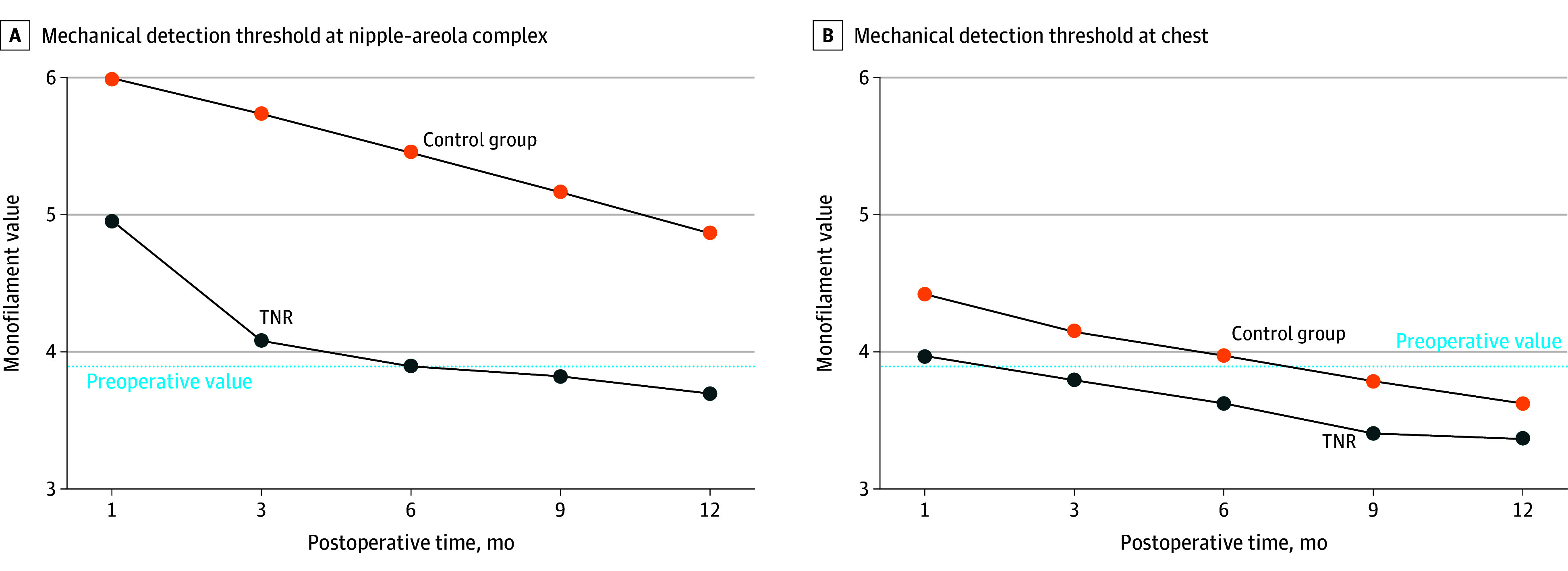
Repeated Measures Analysis of Variance of Mechanical Detection Threshold Over Time A, Association of targeted nipple-areola complex reinnervation (TNR) with the mean mechanical detection threshold over time at the nipple-areola complex, and B, the chest. Blue line represents patients who underwent TNR, while orange line represents patients who did not.

Repeated measures ANOVA showed that the main group association of TNR with mechanical detection threshold was significant over postoperative time at the NAC (*F* = 35.2; *P* < .001) and chest (*F* = 4.2; *P* = .045). In patients who underwent TNR, the mean (SD) thresholds became comparable with preoperative values starting at 3 months postoperatively for the NAC (4.0 [0.7] vs 3.9 [0.5]; *t*_88_ = 1.05; *P* = .30) and 1 month postoperatively for the chest (3.8 [0.6] vs 3.6 [0.6]; *t*_86_ = 1.73; *P* = .09) (see Figure 1). At 12 months, mean quantitative sensory values in patients who underwent TNR reached baseline and were improved compared with patients who did not for monofilaments (mean [SD] NAC, 3.7 [0.5] vs 4.9 [0.9]; *t*_92_ = 7.63; *P* < .001; chest, 3.3 [0.4] vs 3.6 [0.6]; *t*_98_ = 3.24; *P* = .002).

#### Additional Quantitative Sensory Testing at 12 Months Postoperation

The mean (SD) vibration detection threshold at the NAC in patients who underwent TNR was significantly better as compared with those who did not (7.7 [0.4] vs 7.3 [0.4]; *t*_96_ = 6.3; *P* < .001) and preoperative values (7.7 [0.4] vs 7.5 [0.4]; *t*_78_ = 2.5; *P* = .01). The mean (SD) vibration detection threshold at the chest was also significantly better in patients who underwent TNR as compared with those who did not (7.8 [0.3] vs 7.5 [0.3]; *t*_96_ = 5.1; *P* < .001) and preoperative values (7.8 [0.3] vs 7.2 [0.4]; *t*_78_ = 7.9; *P* < .001).

Significantly more patients who underwent TNR could feel 2 point-discrimination at the NAC as compared with those who did not (40% vs 0%; *r* = 20; *P* = .01) and this rate was comparable with preoperative values (40% vs 48%). The mean (SD) 2-point discrimination at the chest in patients who underwent TNR was significantly improved as compared with those who did not (4.1 [1.2] cm vs 5.7 [1.8] cm; *t*_94_ = 5.1; *P* < .001) and preoperative values (4.1 [1.2] cm vs 4.8 [0.7] cm; *t*_78_ = 2.5; *P* = .01).

The mean (SD) pinprick detection threshold at the NAC in patients who underwent TNR was significantly improved as compared with those who did not (24.9 [21.2] mN vs 82.6 [96.7] mN; *t*_98_ = 4.1; *P* < .001) and comparable with preoperative values (24.9 [21.2] mN vs 16.7 [16.9] mN; *t*_70_ = 1.8; *P* = .12). The mean pinprick threshold at the chest in patients who underwent TNR was significantly improved as compared with those who did not (22.5 [25.6] mN vs 54.1 [45.4] mN; *t*_98_ = 4.6; *P* < .001) and comparable with preoperative values (22.5 [25.6] mN vs 17.2 [17.1] mN; *t*_70_ = 0.9; *P* = .38).

All patients who underwent TNR (100%) and 39 who did not (78%) were able to detect cold at the NAC before reaching the maximum threshold of 0 °C (*r* = 12.3; *P* < .001). Patients who underwent TNR could detect cold at the NAC significantly sooner as compared with those who did not (mean [SD], 23.1 [4.7] °C vs 12.0 [7.6] °C; *t*_98_ = 8.8; *P* < .001), and at comparable levels with preoperative values (23.1 [4.7] °C vs 24.6 [4.9] °C). At the chest, all patients who underwent TNR (100%) and all of those who did not (100%) were able to detect cold before reaching 0 °C. Cold detection in patients who underwent TNR was significantly sooner than those who did not (23.6 [3.1] °C vs 19.7 [5.6] °C; *t*_98_ = 4.4; *P* < .001), and comparable with preoperative values (23.6 [3.1] °C vs 23.8 [3.8] °C).

All patients who underwent TNR (100%) and 30 of those who did not (60%) could detect warm before reaching the maximum threshold of 50 °C (*r* = 25; *P* < .001). Patients who underwent TNR could detect warm at the NAC significantly sooner as compared with those who did not (mean [SD], 39.9 [5.0] °C vs 45.8 [4.2] °C; *t*_98_ = 6.3; *P* < .001) and comparable with preoperative values (mean [SD], 39.9 [5.0] °C vs 38.4 [6.4] °C; [data]; *P* = .20). At the chest, all patients who underwent TNR (100%) and 45 who did not (90%) were able to detect warm before reaching 50 °C (*r* = 5.3; *P* = .02). Patients who underwent TNR could detect warm at the chest significantly sooner as compared with those who did not (mean [SD], 39.4 [3.1] °C vs 42.9 [4.0] °C; *t*_98_ = 4.9; *P* < .001) and comparable with preoperatively (mean [SD], 39.4 [3.1] °C vs 38.8 [3.1] °C; [data]; *P* = .32).

All (100%) patients who underwent TNR and 28 who did not (56%) were able to detect heat pain at the NAC before reaching the maximum threshold of 50 °C (*r* = 28.2; *P* < .001). Patients who underwent TNR could detect heat pain at the NAC significantly sooner as compared with those who did not (mean [SD], 46.7 [3.1] °C vs 48.8 [1.8] °C; *t*_98_ = 4.0; *P* = 001), but not as soon as preoperatively (mean [SD], 46.7 [3.1] °C vs 43.0 [3.7] °C; *t*_100_ = 5.4; *P* < .001). At the chest, heat pain could be detected in 100% of patients who underwent TNR and 100% of those who did not. Heat pain was detected significantly sooner in patients who underwent TNR as compared with those who did not (mean [SD], 44.8 [2.2] °C vs 45.8 [2.2] °C; *t*_98_ = 2.1; *P* = .04), but not as soon as preoperatively (mean [SD], 44.8 [2.2] °C vs 43.2 [3.2] °C; *t*_100_ = 2.7; *P* = .01).

**Figure 2.**
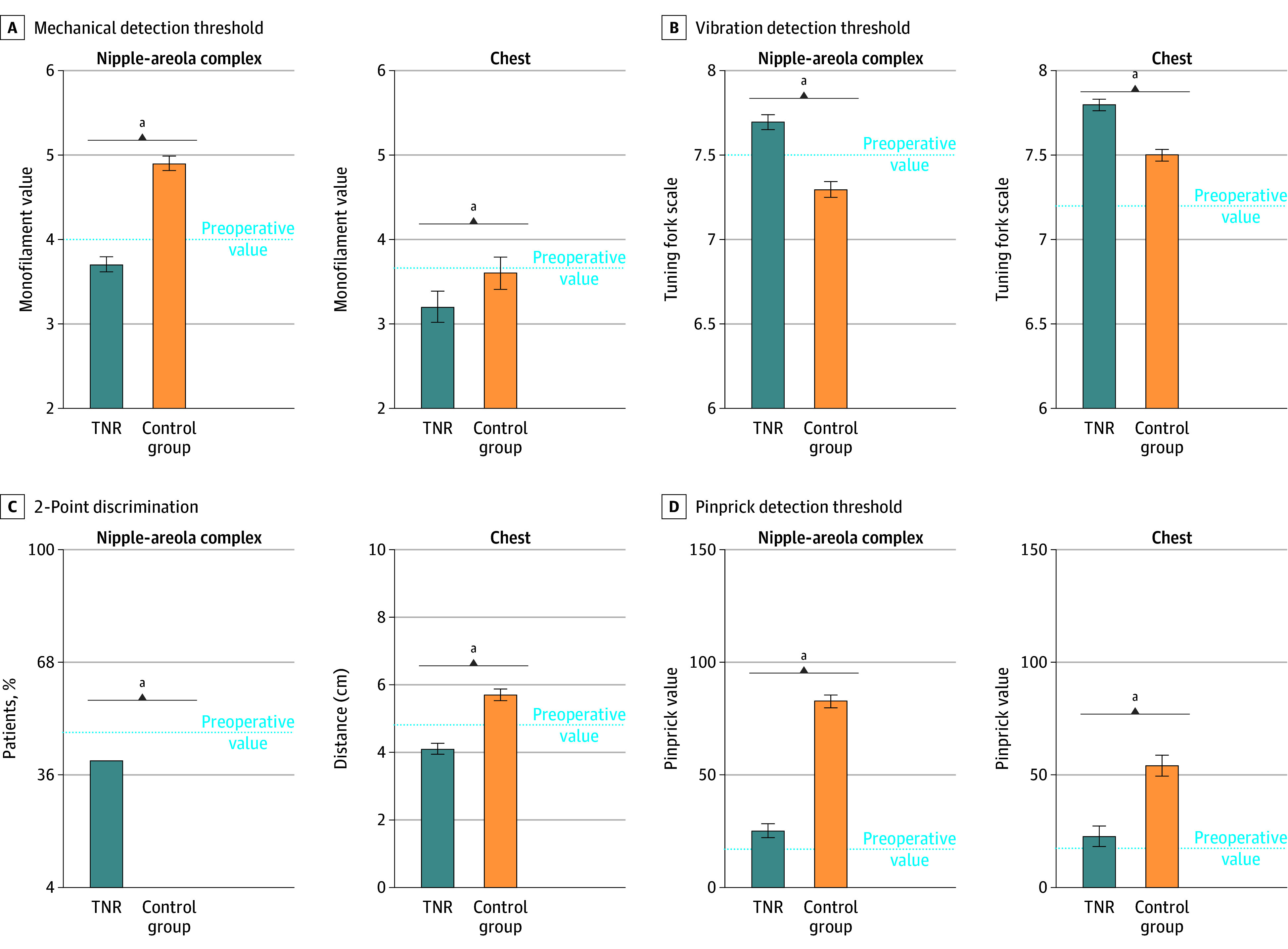
Quantitative Sensory Testing Testing of A, mechanical; B, vibration; C, 2-point discrimination; and D, pinprick at the nipple-areola complex and chest in patients who underwent targeted nipple-areola complex reinnervation as compared with patients who did not. Error bars represent SEs. ^a^Represents a statistically significant difference of *P* < .05.

**Figure 3.**
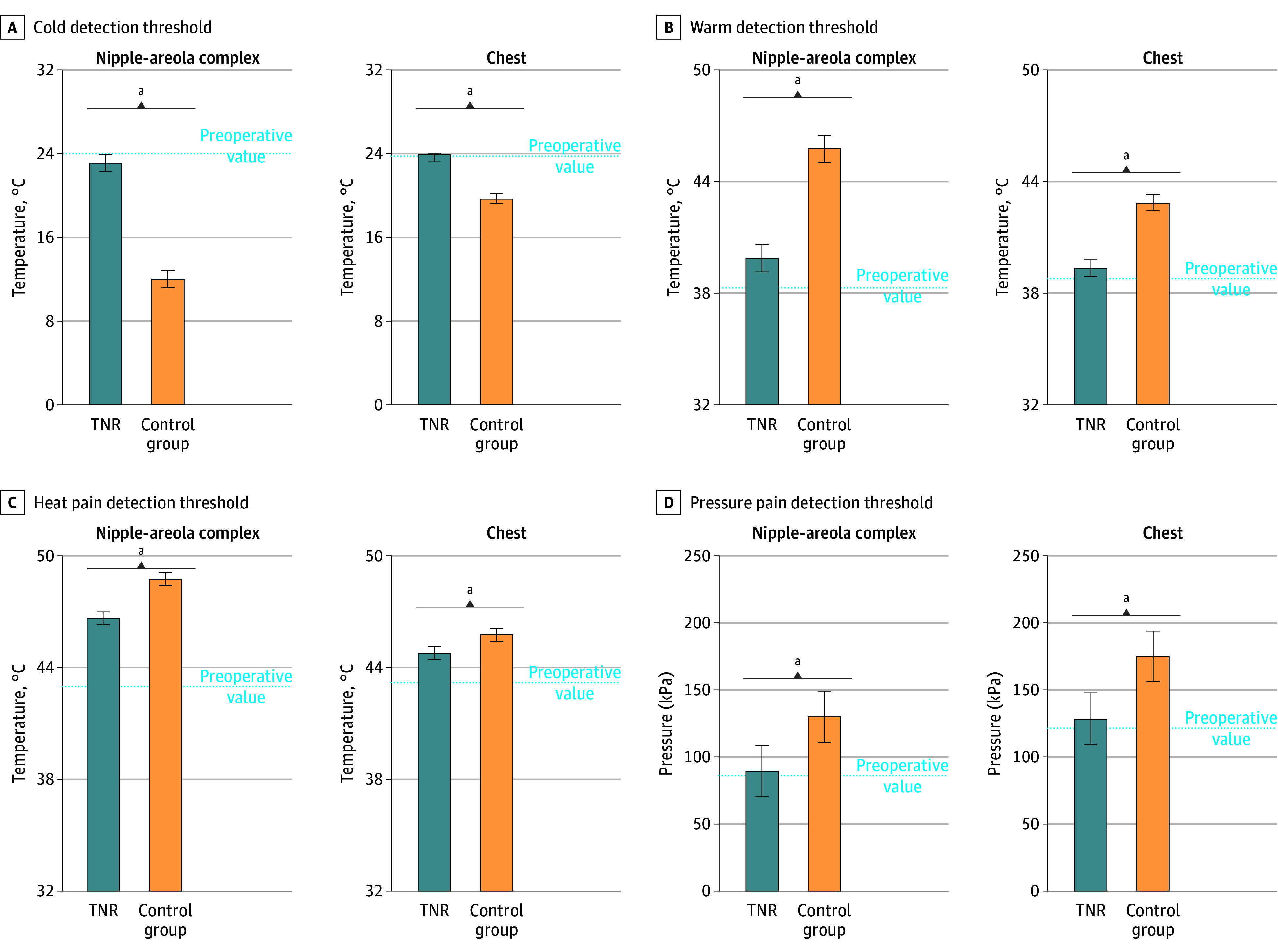
Quantitative Sensory Testing Testing of A, cold; B, warm; C, heat pain; and D, pressure pain detection thresholds at the nipple-areola complex and chest in patients who underwent targeted nipple-areola complex reinnervation as compared with patients who did not. Error bars represent SEs. ^a^Represents a statistically significant difference of *P* < .05.

All patients who underwent TNR (100%) and all of those who did not (100%) were able to detect pain upon application of pressure on the NAC and chest. The mean (SD) pressure pain detection threshold at the NAC in patients who underwent TNR was significantly lower as compared with those who did not (89.9 [45.6] kPa vs 130.5 [68.9] kPa; *t*_86_ = 3.9; *P* < .001) and was comparable with preoperative values (89.9 [45.4] kPa vs 86.1 [73.5] kPa). Values at the chest in patients who underwent TNR were significantly lower as compared with those who did not (mean [SD], 128.5 [38.0] kPa vs 175.5 [49.3 kPa]; *t*_96_ = 4.0; *P* = .001) and comparable with preoperative values (mean [SD], 128.5 [38.0] kPa vs 121.7 [57.7] kPa) (see Figures 2 and 3).

### Patient-Reported Outcomes

**Figure 4.**
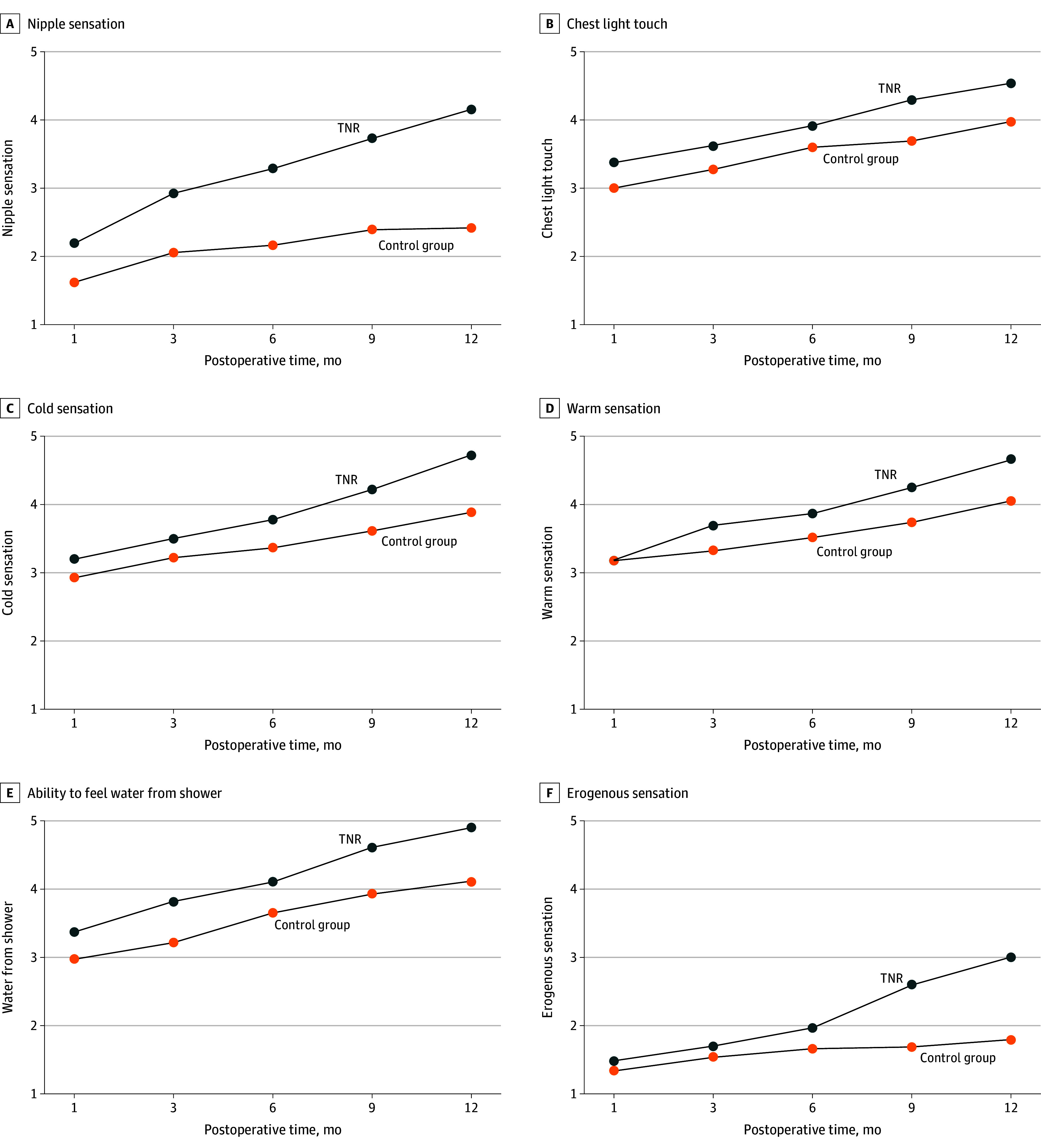
Repeated Measures Analysis of Variance of Patient-Reported Outcomes Over Time Associations of targeted nipple-areola complex reinnervation (TNR) over time with postoperative patient-reported A, nipple sensation; B, chest light touch; C, cold sensation; D, warm sensation; E, the ability the feel water from the shower; and F, erogenous sensation. Patient-reported sensation was rated as either 1 = no sensation, 2 = a little sensation, 3 = some sensation, 4 = a lot of sensation, 5 = complete sensation. Blue line represents patients who underwent TNR, while orange line represents patients who did not.

Preservation of sensation was felt to be important for all patients who underwent TNR and most of those who did not (20 patients [80%]) preoperatively. Repeated measures ANOVA showed that the association of TNR with patient-reported sensation was significant over postoperative time for nipple sensation (*F* = 60.5; *P* < .001), chest light touch (*F* = 8.1; *P* = .01), cold sensation (*F* = 8.1; *P* = .01), warm sensation (*F* = 5.6; *P* = .02), the ability to feel water from the shower (*F* = 9.0; *P* = .004) and erogenous sensation (*F* = 8.3; *P* = .01), but not for chest pressure and the ability to feel a hug (see Figure 4).

At 12 months postoperatively, patients reported being either extremely (25 patients [50%]), very (15 patients [30%]), or somewhat (10 patients [20%]) satisfied with nipple sensation, while control patients reported being either extremely (7 patients [14%]), very (17 patients [34%]), or somewhat (31 patients [62%]) satisfied. For chest sensation, patients who underwent TNR reported being either extremely (27 patients [55%]) or very (23 patients [45%]) satisfied, while those who did not reported being either extremely satisfied (15 patients [30%]), very satisfied (25 patients [50%]), somewhat satisfied (5 patients [10%]), or somewhat dissatisfied (5 patients [10%]). At 12 months postoperatively, 18 patients who underwent TNR (72%) and 5 who did not (20%) reported some degree of nipple erection (*r* = 10.5; *P* = .001).

Nipple hypersensitivity was reported by significantly more patients who underwent TNR than those who did not at 1 and 3 months postoperatively but at comparable rates afterwards. At 12 months postoperatively, 2 patients who underwent TNR (8%) and 1 who did not (4%) reported nipple hypersensitivity. Patients with persisting nipple hypersensitivity at 12 months were successfully treated with desensitization therapy.

### Adverse Events

Adverse events occurred in 5 cases (10%), including 1 control (2%) who required aspiration of a seroma, 3 patients (6%) who developed hypertrophic scarring at the inframammary fold, and 1 patient (2%) who presented with nipple hypopigmentation. There were no cases of partial or complete free nipple graft necrosis. There were no statistically significant differences in the occurrence of adverse events between patients who underwent TNR and those who did not.

## Discussion

To our knowledge, this is the first prospective matched cohort study evaluating the outcomes of sensory reinnervation in gender-affirming mastectomy. Overall, we found that patients who underwent TNR had significantly improved restoration of quantitative as well as patient-reported sensory outcomes compared with control patients who did not undergo TNR. These findings suggest the efficacy of TNR in improving postoperative sensation following gender-affirming female to male chest surgery.

### Quantitative Sensory Outcomes

Patients who underwent TNR were able to preserve nearly all quantitative sensory functions at both the NAC and chest. While baseline values were surpassed for mechanical, vibration, and 2-point discrimination, values reached baseline for pinprick, temperature, and pressure pain thresholds. Only heat pain was not restored, although values were improved as compared with patients who did not undergo TNR. In contrast, patients who did not undergo TNR experienced persistent loss of all quantitative sensory functions at the NAC. At the chest, while some sensory functions, such as mechanical detection, vibration, and 2-point discrimination, were restored in patients who did not undergo TNR, other functions such as pinprick, temperature, heat pain, and pressure pain detection were not preserved.

These findings are important given the possible functional and psychosocial consequences associated with impaired sensation. In patients undergoing cancer mastectomy, postoperative loss of sensation has been shown to increase the risk of injuries including burns from water or thermal packs, interfere with daily activities such as carrying objects, and result in significantly reduced chest physical well-being.^11,24,25^ Given that between 52.4% to 100% of patients undergoing gender-affirming mastectomy lose varying degrees of sensation, the negative implications associated with sensory loss may also apply to this patient population.^6,7,8^

Since certain sensory functions reached or surpassed baseline, the reduction of breast tissue volume that occurs during the mastectomy procedure may facilitate sensory preservation. Reduced tissue volume may increase the nerve density per tissue volume, thus resulting in better sensation. Consistently, reports have shown increased sensitivity with lower mastectomy weights and BMIs as well as improved sensation following breast reduction surgery.^19,26^

Furthermore, the increased sensitivity to mechanical, 2-point discrimination, and vibration as compared with temperature and pain detection could originate from variations in underlying nerve fiber characteristics and reinnervation potential. While mechanical detection, vibration, and 2-point discrimination are regulated by large, myelinated fibers (type Aβ fibers), temperature and pain are mediated by small thinly myelinated or unmyelinated fibers (type Aδ and type C fibers, respectively). Animal studies have suggested that large myelinated fibers undergo faster and more complete reinnervation and may be more resistant to nerve injury as compared with small unmyelinated fibers.^27,28^ However, this field has been largely unexplored.

### Patient-Reported Sensory Outcomes

The patient-reported outcomes associated with TNR were found to correspond to the quantitative findings, with significant improvements in patient-reported nipple sensation, chest light touch, and temperature sensation. Interestingly, erogenous sensation and nipple erection were also improved in patients who underwent TNR. Although the specific sensory fibers and distal receptors responsible for erogenous sensation are unknown, autonomic fibers within the intercostal nerves innervating the NAC may have a role.^29^ Similarly, nipple erection may also be regulated by autonomic nerves innervating the smooth muscle fibers within the NAC.^30^ The nipples and chest are important anatomic zones for erogenous and sexual function in both males and females.^12,31^ Several studies have shown that preservation of nipple and chest sensation following cancer mastectomy and breast reconstruction is associated with improved sexual well-being.^32,33^ Therefore, improving sensory outcomes may similarly benefit the sexual well-being of patients undergoing gender-affirming mastectomy. The significant improvements in the various patient-reported sensory functions in patients who underwent TNR likely explains the overall higher satisfaction rate for both nipple and chest sensation in these patients.

Lastly, TNR was found to be associated with a higher risk of transient nipple hypersensitivity until 3 months postoperatively. This risk can be minimized by splitting the intercostal nerve fascicles distally underneath the free nipple grafts to increase the reinnervation surface area.^18^ Patients should be counseled about this risk and treated with desensitization therapy if necessary.

### Limitations

The results of this study should be acknowledged within the context of its limitations. First, while mechanical detection threshold assessed with monofilament testing was the primary outcome variable, other analyses should be interpretated as exploratory. Second, bias may have been introduced due to the self-selection process of patients undergoing TNR. Third, we did not assess patient-reported outcomes regarding psychosocial well-being. Additionally, our study was limited by a follow-up duration of 12 months.

## Conclusions

In this prospective matched cohort study of patients undergoing double-incision gender-affirming mastectomy with free nipple grafting, TNR was associated with improved restoration of quantitative and patient-reported mechanical, temperature, and pain detection, as well as erogenous sensation at the NAC and chest. Patients undergoing TNR should be counseled about the risk of transient NAC hypersensitivity.

## Notes

Supplement 1. **eFigure 1. **Double-Incision Gender-Affirming Mastectomy With Free Nipple Grafting and Targeted Nipple-Areola Complex Reinnervation **eFigure 2. **Quantitative Sensory Testing Performed on Predefined Quadrants of the Nipple-Areola Complex and Chest **eTable.** Full Verbatim Text of Questions Used for Patient-Reported Outcomes

Supplement 2. Data Sharing Statement
