# Supplementary material for: Targeted Reinnervation During Gender-Affirming Mastectomy and Restoration of Sensation
Source: JAMA Netw Open. 2024 Nov 22;7(11):e2446782. doi: 10.1001/jamanetworkopen.2024.46782 (PMC11584927; doi:10.1001/jamanetworkopen.2024.46782)
Supplement: Supplement 1. — eFigure 1. Double-Incision Gender-Affirming Mastectomy With Free Nipple Grafting and Targeted Nipple-Areola Complex Reinnervation eFigure 2. Quantitative Sensory Testing Performed on Predefined Quadrants of the Nipple-Areola Complex and Chest eTable. Full Verbatim Text of Questions Used for Patient-Reported Outcomes [file jamanetwopen-e2446782-s001.pdf]

Supplemental Online Content

Remy K, Alston C, Gonzales E, et al. Targeted reinnervation during gender-affirming mastectomy with restoration of sensation. *JAMA Netw Open*. 2024;7(11):e2446782. doi:10.1001/jamanetworkopen.2024.46782

eFigure 1. Double-Incision Gender-Affirming Mastectomy With Free Nipple Grafting and Targeted Nipple-Areola Complex Reinnervation

eFigure 2. Quantitative Sensory Testing Performed on Predefined Quadrants of the Nipple-Areola Complex and Chest

eTable. Full Verbatim Text of Questions Used for Patient-Reported Outcomes

This supplemental material has been provided by the authors to give readers additional information about their work.

© 2024 Remy K et al. *JAMA Network Open.*

eFigure 1. Double-Incision Gender-Affirming Mastectomy With Free Nipple Grafting and Targeted Nipple-Areola Complex Reinnervation (TNR). The various steps of the procedure are illustrated.

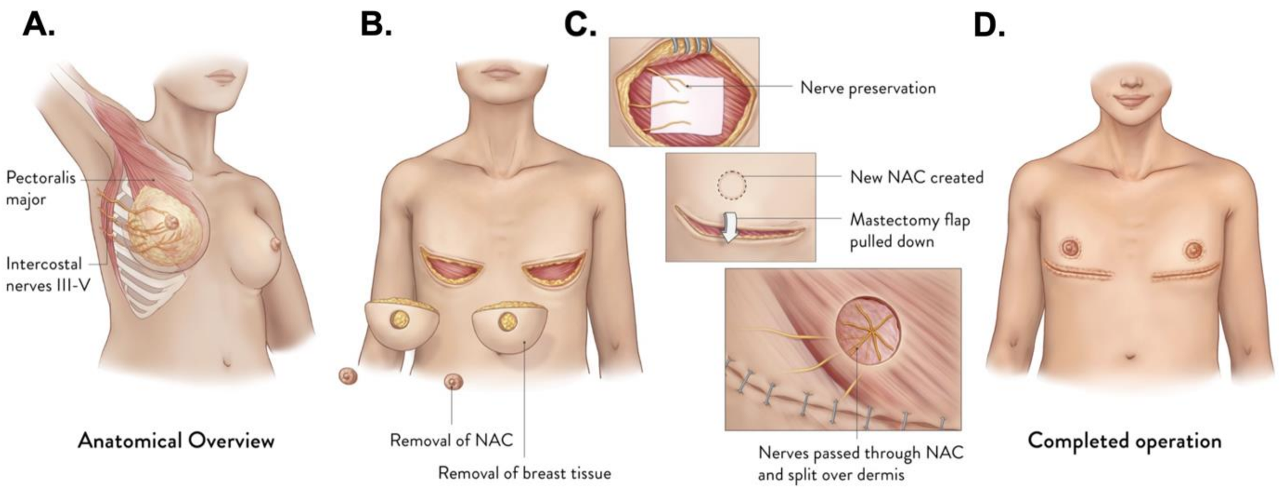

eFigure 2. Quantitative Sensory Testing Performed on Predefined Quadrants of the Nipple-Areola Complex and Chest

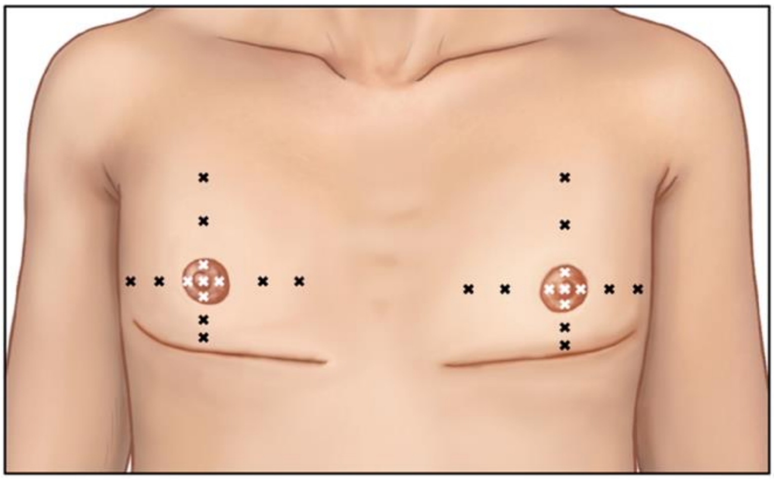

eTable. Full Verbatim Text of Questions Used for Patient-Reported Outcomes

© 2024 Remy K et al. *JAMA Network Open.*

How concerned are you with nipple sensation?	1. Not at all concerned<br>2. Slightly concerned<br>3. Moderately concerned<br>4. Very concerned<br>5. Extremely concerned
How concerned are you with sensation of the chest skin?	1. Not at all concerned<br>2. Slightly concerned<br>3. Moderately concerned<br>4. Very concerned<br>5. Extremely concerned
How concerned are you about protective sensation (for example ability to feel hot/ cold so you don't get burned)?	1. Not at all concerned<br>2. Slightly concerned<br>3. Moderately concerned<br>4. Very concerned<br>5. Extremely concerned
How much feeling do you have in your right nipple?	1. No feeling<br>2. A little feeling<br>3. Some feeling<br>4. A lot of feeling<br>5. Complete feeling
How much feeling do you have in your left nipple?	1. No feeling<br>2. A little feeling<br>3. Some feeling<br>4. A lot of feeling<br>5. Complete feeling
Are you able to feel light touch on your right chest?	1. No feeling<br>2. A little feeling<br>3. Some feeling<br>4. A lot of feeling<br>5. Complete feeling
Are you able to feel light touch on your left chest?	1. No feeling<br>2. A little feeling<br>3. Some feeling<br>4. A lot of feeling<br>5. Complete feeling
Are you able to feel pressure on your right chest (such as massaging your chest deeply)?	1. No feeling<br>2. A little feeling<br>3. Some feeling<br>4. A lot of feeling<br>5. Complete feeling
Are you able to feel pressure on your left chest (such as massaging your chest deeply)?	1. No feeling<br>2. A little feeling<br>3. Some feeling<br>4. A lot of feeling<br>5. Complete feeling
Are you able to feel cold on your right chest?	1. No feeling<br>2. A little feeling<br>3. Some feeling<br>4. A lot of feeling<br>5. Complete feeling
Are you able to feel cold on your left chest?	1. No feeling<br>2. A little feeling<br>3. Some feeling<br>4. A lot of feeling<br>5. Complete feeling
Are you able to feel warm on your right chest?	1. No feeling<br>2. A little feeling<br>3. Some feeling<br>4. A lot of feeling<br>5. Complete feeling

© 2024 Remy K et al. *JAMA Network Open.*

Are you able to feel warm on your left chest?	1. No feeling<br>2. A little feeling<br>3. Some feeling<br>4. A lot of feeling<br>5. Complete feeling
Are you able to feel water from the shower on your right chest?	1. No feeling<br>2. A little feeling<br>3. Some feeling<br>4. A lot of feeling<br>5. Complete feeling
Are you able to feel water from the shower on your left chest?	1. No feeling<br>2. A little feeling<br>3. Some feeling<br>4. A lot of feeling<br>5. Complete feeling
Are you able to feel a hug?	1. No feeling<br>2. A little feeling<br>3. Some feeling<br>4. A lot of feeling<br>5. Complete feeling
How much erogenous sensation do you have if your right nipple is touched sexually?	1. No feeling<br>2. A little feeling<br>3. Some feeling<br>4. A lot of feeling<br>5. Complete feeling
How much erogenous sensation do you have if your left nipple is touched sexually?	1. No feeling<br>2. A little feeling<br>3. Some feeling<br>4. A lot of feeling<br>5. Complete feeling
Has your right nipple conserved the ability to become erect (such as when you are feeling cold or during erogenous sensation)?	1. Not at all<br>2. A little bit<br>3. Quite a bit<br>4. Very much
Has your left nipple conserved the ability to become erect (such as when you are feeling cold or during erogenous sensation)?	1. Not at all<br>2. A little bit<br>3. Quite a bit<br>4. Very much
On a scale of 0-10, how much hypersensitivity do you have in your right nipple?	
On a scale of 0-10, how much hypersensitivity do you have in your left nipple?	

© 2024 Remy K et al. *JAMA Network Open.*
