# Supplementary material for: Targeted Reinnervation During Gender-Affirming Mastectomy and Restoration of Sensation
Source: JAMA Netw Open. 2024 Nov 22;7(11):e2446782. doi: 10.1001/jamanetworkopen.2024.46782 (PMC11584927; doi:10.1001/jamanetworkopen.2024.46782)
Supplement: Supplement 2. — Data Sharing Statement [file jamanetwopen-e2446782-s002.pdf]

Data Sharing Statement

Remy. Targeted Reinnervation During Gender-Affirming Mastectomy With Restoration of Sensation. *JAMA Netw Open*. Published November 21, 2024.
doi:10.1001/jamanetworkopen.2024.46782

Data
Data available: Yes
Data types: Deidentified participant data
How to access data: kremy11@mgh.harvard.edu
When available: With publication

Supporting Documents
Document types: Statistical/analytic code
How to access documents: kremy11@mgh.harvard.edu
When available: With publication

Additional Information
Who can access the data: Any qualified researcher
Types of analyses: Any purpose
Mechanisms of data availability: With investigator support
